# HIV and gender identity expression among transfeminine women in the Western Cape, South Africa – a thematic analysis of data from the HPTN 071 (PopART) trial

**DOI:** 10.21203/rs.3.rs-2486896/v1

**Published:** 2023-02-06

**Authors:** Laing De Villiers, Leslie Swartz, Peter Bock, Janet Seeley, Anne L. Stangl, Virginia Bond, James Hargreaves, Graeme Hoddinott

**Affiliations:** Stellenbosch University; Stellenbosch University; Stellenbosch University; London School of Hygiene & Tropical Medicine; Johns Hopkins Bloomberg School of Public Health; London School of Hygiene & Tropical Medicine; London School of Hygiene & Tropical Medicine; Stellenbosch University

**Keywords:** transgender, gender identity, HIV, gender expression, South Africa

## Abstract

**Introduction:**

Transfeminine women in South Africa have a high HIV risk due to structural, behavioural, and psychosocial factors. Transfeminine women and feminine identifying men who have sex with men (MSM) are often conflated or grouped with transgender or MSM categories in HIV service programming, although they don’t necessarily identify as either. We aimed to investigate gender expression among feminine identifying people who were assigned male at birth. We examined how local conceptualizations of sexuality and gender intersect with the key population label of ‘transgender’ imported into local HIV programming.

**Methods:**

A qualitative cohort nested within the HPTN 071 (PopART) trial included longitudinal, in-depth interviews with eight transfeminine women (four who disclosed as living with HIV). Data were collected approximately every six weeks between January 2016 and October 2017. We discuss gender identification presented in participants’ daily lives and in relation to HIV service access.

**Results:**

Of the eight participants, only one accepted ‘transgender’ as a label, and even she used varying terms at different times to describe her identity. For participants, a feminine identity included dressing in normatively feminine clothes; using feminine terms, pronouns and names; and adopting stereotypically feminine mannerisms. Participants would switch between typically feminine and masculine norms in response to contextual cues and audience. For example, some participants accepted identification as masculine gay men amongst their family members, but amongst peers, they expressed a more effeminate identity and with partners they took on a feminine identity.

**Conclusions:**

Our findings are amongst the first exploratory and descriptive data of transfeminine women in South Africa. We show how transfeminine women navigate fluid gender identities that could pose a challenge for accessing and utilizing HIV services that are currently set up for transgender individuals or MSM. More work needs to be done to understand and respond to the diverse and shifting ways people experience their gender identities in this high HIV burden context.

## Introduction

Over the past six years, heightened focus has been placed on transgender women in HIV research worldwide [1, 2]. ‘Transgender’ is an umbrella term conventionally used to describe someone who identifies as a different gender than their biological assigned sex at birth [3, 4], which also includes a variety of gender diverse and possibly non-binary individuals with a range of gender expressions and sexual partner preferences. In some contexts, the term ‘transgender’ is predominantly used to describe people who have accessed or intend to access gender transitional health services to shift their biology to reflect their self-identity [5]. As such, the term ‘transgender’ is often connected to a fixed gender identity, potentially obscuring experiences of gender as fluid [4]. Indeed, the majority of HIV research with the transgender community to date has focused on fixed gender identity [1].

In this paper, we use the term ‘transfeminine’ to refer to people assigned male at birth who identify as women in major parts of their lives but have fluid ways of expressing their gender identity [6]. Transgender women have been recognised as a group at high risk of HIV due to structural, behavioural, and psychosocial risk factors and are often assumed to have specific health needs (i.e. transitional surgery, hormonal treatment) when accessing healthcare [2, 7, 8]. However, people who may be classified as transgender women include a broad range of gender diverse people and transfeminine women who were biologically assigned male sex at birth [5, 9], some of whom identify as feminine in some ways, and many who have no intention to transition or align their sexual identity and gender identity.

In HIV service programming, transfeminine women are often categorized as transgender women, gay men or MSM [9]. Conflation of people who have trans and diverse gender identifications happens in HIV research and programming, where transgender women and MSM are two ‘at-risk’ population categories [10, 11]. According to Poteat et al. [12], the Conflation of these categories is not only inaccurate but can also pose a harmful risk in that it does not distinguish the unique experiences and sources of risk and vulnerability among transgender individuals, which require specialised interventions.

In a recent study, Brumbaugh-Johnson et al. [13] used a gender theory lens to show that gender expression and disclosure among transgender people is a complex process based on social enactment and circumstances. For example, in the South African, Western Cape coloured community context, taking on hyper feminized roles by feminine identifying gay men and transfeminine women helped to regulate and counteract predominant hegemonic masculine discourse and behaviour [14]. Sandfort [15] explains: “to be gendered implies far more than being allocated some neutral and taken-for-granted label but is to be subject to a range of regulatory mechanisms and moral prohibitions.” Because gender is done in social interactions, trans people find appropriate ways of feminine and masculine gender expression, according to certain situations and with different people [16] as a way of fitting in, surviving and protecting themselves from being stigmatized [9].

Very little is known about the experiences of transfeminine women in South Africa, a country that accounts for approximately one fifth of all people living with HIV in the world [17]. We aimed to show the complexity of transfeminine women’s experiences in a high HIV-burden area, the Western Cape of South Africa. Specifically, we a) describe the different terms participants use to describe their gender identity and gender expression; and b) present case examples of how gender identity is expressed differently for these women depending on their contexts.

## Methods

### Study design

This was an exploratory study using secondary data from a qualitative cohort. We made use of a mixed methods approach combining thematic analysis and case study descriptions. Data used for this study were accessed from a sub-sample of a qualitative cohort study nested in the HPTN 071 (PopART) trial. As part of the nested social science evaluation, we conducted qualitative research with a cohort of approximately 90 families spread across nine study communities in the Western Cape, South Africa. Eight of these families included at least one transfeminine woman (data analyzed here).

### Recruitment

We approached a variety of community members; clinic staff; trial intervention staff; Lesbian, Gay, Bisexual, Transgender, Queer and Intersex (LGBTQI) community members; and men’s health clinic staff to identify potential participants. Through this process, thirteen transfeminine women were initially approached for participation, five of whom were lost to follow up or opted out of participation in the study. Of the remaining eight, four disclosed to us that they were living with HIV.

### Data collection

Data collection was informed by ethnographic and participatory research principles. Our team recorded the discussions with voice recorders and semi-structured field notes and took pictures of relevant activities. All interactions with participants happened *in situ* in study communities – usually in participants’ homes, but also often as they moved about the study community completing their daily activities. Researchers interacted with households for several hours per interaction and multiple times over the course of the data collection period, which took place from January 2016 to October 2017. Apart from recorded interviews, we also collected data via field notes, reflection documents, photographs and participatory research activities (e.g., drawing a map of the community with the participants) during visits. Data collection was done by pairs of graduate social science researchers. The first author (LdV) was one of the researchers who visited all eight participants involved in this analysis and transcribed most of the interview data. We would often do extended visits with the participants, walking around in their community, accompanying them to clinic visits and modelling/drag show events. Due to the sensitive nature of the work and spending time in people’s personal lives, we found that the longitudinal nature of the engagement helped build rapport between researchers and participants. In addition, the strong connections developed with participants allowed us to manage the discussion of events in their lives which were traumatic, and might re-invoke trauma, sensitively. In total, discussions and observation activities with the eight participants and their household members took place over 146 hours, of which 82 hours of interviews were with the transfeminine women.

### Data analysis

We conducted a thematic analysis of the terms and concepts used by participants related to their gender identification. This was an open coding process, done with NVivo 12 (QSR International) software. These themes are presented in Table 1. We then provided case descriptions for the different participants to show negotiation of gender identity terms in different contexts. We initially developed larger narratives about each participant – including sub-sections about their house (physical) and household membership structure, employment, social and other life experiences. From the larger narratives, short case descriptions were then developed to show how gendered terms and identifications were dealt with by participants on a daily and lived basis and how these terms and identities possibly influenced their HIV care.

### Ethical considerations

All participant household members signed consent forms to have their households participate in the qualitative cohort. The consent also expressed research outside of the households and documents various other parts of the participants. For any events and activities researchers ensured participant willingness. Each household member also had a personal choice to participate or not. Participants were informed during the consent process about the possible sensitive nature of the interviews. Pseudonyms are used here for each participant, family members, neighborhoods and towns to protect confidentiality. Participants were not given incentives. Instead, research staff received allowances of approximately 80 South African Rands (about 6 USD) per visit to share household costs, contribute to shared meals, and/or gifts for participants and their households.

## Findings

For participants, expressing their ‘feminine identity’ included dressing in normatively feminine clothes, using feminine terms, pronouns and names, as well as other feminine attributes and mannerisms. Participants’ gender identity and expression changed often in the way they referred to themselves and how others referred to them in different contexts. We first provide a descriptive table of terms that participants and people used throughout the study for referring to their gender identity. We then present case descriptions as examples to illustrate how gender was navigated by different participants in different contexts.

### Description of terms participants use to describe their gender identity and gender expression

Participants used a variety of gender identity expressions and ways of referring to themselves. These terms and gender expressions were, however, context bound, referring to people, time and place. Table 1 shows the terms used by participants as well as situations where their gender identity might be expressed in a more masculine and feminine manner.

The table illustrates pseudonym birth given names of the participants (which relate to their masculine biological assigned sex at birth) and then some participants used self-assigned names and nicknames connected to their feminine identity. All names here are fictional pseudonyms that illustrate this pattern. For example, Simone’s birth given name is Owen Andrews, but she has chosen the name Simone as it more accurately connects to her feminine gender identity. Alongside gender-typical names, we show a variety of terms used by participants throughout the study in their interviews. These are terms that others might use to refer to them or ones that they use to refer to themselves. The term “moffie” for example is a derogatory Afrikaans term used to refer to effeminate gay men, with possible origins from the Dutch “mofrodiet” for ‘hermaphrodite’ [18, 19]. Most of the participants used the word as a self-claimed term to refer to themselves and their friends and peers. ‘Moffie’ is also often used by community members to address the transfeminine women we interviewed and their peers. Most of the terms used by participants are feminine identifying terms, like femgay, queen, woman or feminine, but they also sometimes used terms related to their biological assigned male sex at birth like man, guy and male. Some terms also had particular reference to their sexual orientation or sex role, like bottom (referring to their preference as receptive anal sex partners) or gay. The table also portrays situations where participants used either feminine or masculine identities depending on the situation. For example, Simone explained that she used feminine gender identity expression terms amongst transfeminine and gay peers and with her romantic partners, whereas her family members (e.g. sister, cousins and aunts) typically referred to her in a masculine way by using her birth given name, Owen.

### Case examples of how gender identity was expressed differently for these women depending on their contexts

24-year-old Simone Owen Andrews is from Town D, a mixed metro and low-income community in the Western Cape. Simone is living with HIV and identifies with the term “transgender woman”, however she also uses the term “gay”. Growing up she always felt and behaved in a feminine manner, playing mostly with girls, cross-dressing, and in this way of being, she told us that people could distinguish that she was not a heterosexual boy. She often uses the term ‘gay’ to refer to herself. Some people still call her by her masculine birth name, Owen, which could potentially mean her having to negotiate a masculine component of her identity (that in some ways she is still seen as a man by some people). Below is an excerpt from an interview in April 2017 where she uses a ‘gay’ identity term:

Researcher okay but then how do you see yourself? You know if they talk about LGBT- or a range of categories, what, how do you categorise yourself?

Simone for now it is “gay”

Researcher just gay?

Simone yes

For Simone and most of the study participants, their feminine gender identity was present from a young age and they have seen it develop over time as they started dressing in feminine clothes, participating in drag/modelling shows and being the woman in their romantic and sexual relationships.

Stacey (born Benjamin Martins) is a 29-year-old self-identified ‘femgay’ individual also living with HIV. She lives in an informal community at the outskirts of a winelands farming town in the Western Cape. Stacey does not live with her immediate family. She lives a very mobile/transient lifestyle, moving between different houses in the immediate neighbourhood and relying on extended family members, friends and other femgay peers to support her with a place to stay, food and other necessities. Even though Stacey might fall under the transgender umbrella as a transfeminine woman, she actually took offence when in an early interview the interviewer mistakenly labelled her gender identification as transgender, as seen in the excerpt below in June 2016:

Researcher We have a lot of things we are interested in, in sex and love and like how many partners people have had and like where does a person meet, like that’s very interesting for me, especially if a person’s transgender, like

(Stacey interrupts her)

Stacey Me? transgender? (As her voice squeaked in disbelief and she seems to be taken aback).

(Researcher tries to regather her words, but Stacey continues)

I’m femgay (she proudly responds)

Curella (Conry Jenkings), a 28-year-old transfeminine woman, also identifies as ‘femgay ‘. She lives outside the Cape Town metropole with her mother, sister and her mother’s boyfriend. Her family and extended family, living with her, address her as Conry and it’s also how she refers to herself. However, dressing and being a woman is how she feels most comfortable and one of the places that she gets to be her full, cross-dressing and feminine self is at cross-dressing modelling shows. Her modelling and feminine name is Curella. She explained her ‘femgay’ identity below (August 2016):

Researcher what is a drag queen?

Conry a drag queen is similar to us in that they also do shows (pause) like put on dresses, and like heels and stuff like that (pause) and then you get

Researcher but is a drag queen necessarily someone who is gay?

Conry yes … and you have them, they are gay but he doesn’t put on women’s clothes, he wears normal men’s clothes

Researcher and so you would distinguish between the two?

Conry yes you get uhm femgay and then you get (drag queens)

Steven Jansen lives in a small wooden house, which is in the backyard of her aunt’s house, with her mother, father and sister, who all view her as a gay man. Steven, like most of the women we interviewed, used a variety of terms to refer to her gender and sexual identity, including gay, femgay and ‘moffie’. Steven has also navigated her gender identity from a young age and even though she is more assured of her feminine gender identity, it still takes navigation in different spaces as she explains about going out socialising at night:

Now a lot of them have made the excuse that “you are more of a woman than … women really are”. That is their excuse … like last night I was sitting in company … then a guy brought me water and I told him, “ah you are too sweet”, and gave him a kiss on the cheek and then he told me, “Jesus!” and his friend said, “God, you do everything just like a real woman, just like a real woman is supposed to do”. (Steven, May 2017)

Sizwe identifies as MSM but uses feminine pronouns and terms to refer to herself. For Sizwe, her gender identity expression, or the way she has to navigate it, is also influenced by cultural significance associated with spaces, as she is expected as a biological male to go through traditional rites of passage and circumcision into manhood. She explained in a later interview that if she were to go through initiation that it wouldn’t be for herself. She would do it purely for cultural obedience. She explicitly stated that she wouldn’t comply with some of the post-initiation observances, like dressing in a jacket and hat. Similarly, when men get together for traditional ceremonies they will sit together in a fenced-off area called a “kraal”. Below is her reaction to the researcher in explaining her gender role amongst men in the kraal:

Researcher okay and then now when you have to sit in the kraal will you sit then with other men maybe?

Sizwe if I am going to sit, I will arrive in the kraal, my dear, but otherwise I won’t sit long. I won’t continuously … what do I want in the kraal?

Researcher it’s a man among men there

Sizwe I am so weak (hinting at her feminine traits)

Researcher you would have also been a man according to culture

Sizwe you can see I am weak … oh no I am not a man you see me dear

(November 2016)

Girlie Gregory Jones and Patricia Arends are two transfeminine women who do sex work. Sex work for them is not a choice, but something they were both coerced into and stayed in as a means of survival. Their navigation of their gender identities was fraught with even more challenges and problems as they have to deal with coercive gangsters who force them into sex work, as well as clients with whom they have to navigate their gender identity.

Below, Girlie expresses the confusion with which she doesn’t actively seem to have to consider whether she is a man or woman; but the realisation that she has aspects of both, even though she identifies more as a woman:

I just am the way I am, see? I am a man but I like dressing like this … I know I am a man but I feel like a woman, do you see? … I just am like this.

(August 2017)

## Discussion

We found that transfeminine women use a variety of ways to express their gender identity. Although all the participants identified as women and in a feminine way, they used a variety of feminine and masculine terms/concepts to refer to themselves in different contexts, depending on spaces/places, time and who they were with. This variety was reflected in interactions in their personal lives, amongst peers, friends, family, and partners, as well as in social interactions with community members, and when accessing services and other formal processes where identification documentation is requested or required.

Globally, people who were born male but have a feminine gender identification may use a range of gender identities like ‘fairies’, ‘queens’, ‘feminine gay’, cross-dressers and transsexuals [5, 20]. In different parts of the world, so-called feminine males have a range of gender identifications embedded in culture and language: *bakla* or *kathoey* in Thailand, *Travesti* in central- and south-America, *Hijras* in India and *Waria* in Indonesia for example [3, 20]. A special issue of Global Public Health in 2016 emphasized a renewed call for a balanced understanding of sexual and gender diversities aimed toward re-evaluating approaches to prevention and health promotion [11]. Local South African researchers have identified similar gaps in HIV research, as they acknowledge the need for deeper investigation into diversity, fluidity and complexity of gender expressions among MSM and transgender women especially when it comes to HIV risk and service access [21–23].

It is evident that gender expression is contextual to the lived reality of a transgender person. As Brumbaugh-Johnson [24] found, transgender people’s expression and disclosure of their gender identity is navigated by expectations and reactions of others, particularly in avoiding potential harmful situations. Trans people for example in the UK and Portugal self-regulated their gender expression and avoided specific spaces perceived as unsafe [16]. In particular, transgender people’s gender identity displays and expression need to be contextualized in time and space [16], similar to findings we found in our study. Recent research has shown how public and private spaces are navigated by trans people to find safe space for them to openly express their gender identity and avoid more stigmatizing ones where they may experience discrimination [25–27]. The private home space is not necessarily more or less of a vulnerable space for trans people than the public and communal space as, for example, certain areas in the community might be avoided and other not, especially at certain times. Or that during certain times a trans person might feel less accepted at home if there is a family member who visits who has trans- or homophobic beliefs.

Participants in our study similarly used a range of terms that were contextualised in time and space. For example, participants wouldn’t use the term gay to identify themselves if their family and community members didn’t use the term, or they would not have adopted an originally denigrating term like “moffie”, if it wasn’t freely used to criticize and discriminate against them. The way that transfeminine women navigate use of terms for gender identity is, as we’ve seen with a variety of global indigenous and locally used terms, very important to how they potentially access health services and other services that require the use of their identification document. Previous research, similar to our results, have pointed to how trans people had to navigate their gender identity in private and public institutions, like home affairs, the workplace, banks, schools and more [16].

In a range of recent South African studies, HIV researchers have been interested in the liminal group of women who fit into the transgender category, like feminine-identifying MSM [28, 29], gender non-confirming MSM [30–32], non-binary and trans-women [33]. Some terms or ways of identifying include “cross-dressing”, “feminine identifying”, “drag queen”, “being trapped in men’s bodies”, and “gay women” [22, 23, 33]. A study by Sandfort et al. [34] showed a variety of colloquial terms used by feminine identifying MSM and transfeminine women in the Tshwane province of South Africa, including: *Stabane*, *stuzana* or *gemmi* (for gay men, demeaning); *trasi*, *skezonke* (men in the wrong body, persons having a penis and a vagina); *Maho*, *magene* (men who are bottoms); *moffie, watermuis, tarashushu* (feminine gay men).

We show in a recent publication, where we used the same sample and data, that gender identity was often conflated with sexual identity for these transfeminine women in the Western Cape, South African context [9]. In this Conflation of gender and sexual identity from other people towards transfeminine women, stigma intersected with other social identities like being a sex worker, using drugs, living with HIV and more [9]. In that study we found that this complicated the way these women accessed HIV services (including testing, treatment, and adherence support).

Despite some progress to further HIV research among transgender populations globally, very little research on the topic has been done in South Africa [35]. From the local terms and concepts from other global studies (Hjira, Travesti, two-spirited and other transgender groups) [3, 8], we suggest that similar exploratory work is urgently needed to better understand South African sexual and gender minority individuals, in particular transfeminine women, and answer questions such as: How do transgender identities intersect with HIV service access. Could transgender-specialized services potentially exclude transfeminine women who don’t use trans language and who have no interest in gender reassignment, hormonal and other transitional treatment and information? What are some of the other gendered terms used by transfeminine and feminine identifying MSM and gay men in South Africa? These are some of the questions we think nuance how health services and programs for transgender people can be better developed and be supportive of local concepts in the South African context.

Strengths of our study are the rich data we collected, using a wide range of data collection methods, about the lived experiences of participants, which enabled us to understand the layers of stigma and trauma transfeminine women experience, how they cope with these stresses, and how they view their gender identity. In addition, the longitudinal nature of the data allowed us to confirm information about especially sensitive topics that take time to unpack as trust was built in the research relationship. Limitations to extrapolation from our findings include: Firstly, our sample size was small. Secondly, the people identified as transfeminine in our study were difficult to find, interview, and retain. Often, transfeminine participants were socially marginalised, moving around a lot and experiencing many interruptions to their daily routines. This may mean that the experiences of participants retained in the study may not be representative of all transfeminine women in the area (e.g., five participants were lost to follow-up). However, our study draws strength from being the first exploratory study of transfeminine women in the Western Cape.

## Conclusions

In this study we found a variety of terms that South African transfeminine women use to describe their gender identity. These terms and concepts are largely contextually bound and transfeminine women in the study, even though all eight participants identified in a feminine way, often used a variety of feminine and masculine gender identifying terms depending on different contexts. These insights can be used by HIV service providers to better meet the needs of this unique population. In the context of HIV service access, this means asking about and accepting people’s gender identity and expression, instead of assuming or imposing a category, like ‘transgender’, and tailoring service delivery to the unique needs of gender fluid populations. In addition, our findings will help to inform the development of gender-responsive HIV prevention, care and treatment services to support countries in achieving the societal enabler targets and global HIV goals by 2030 [36].

## Figures and Tables

**Table 1.** Gender identity and expression terms used by participants

Pseudonym	Words participant uses to refer to gender identities (all, not just their preferred label)	Situations where participant identifies in feminine ways	Situations where participant identifies in masculine ways
Simone Owen Andrews	*skeef*(skew/not straight), *moffie*, feminine gay, transgender, gay, *vroumens* (woman), feminine pronouns, *meisie* (girl), drag	Around friends and LGBTQI peers; with sexual and romantic partners	With family (sister), extended family (aunts, cousins) heterosexual friends (in these instances others as well as herself use her masculine name, Owen)
Steven Jansen	Daughter/woman, *moffie*, femgay, cross-dresser, woman, man (limited use), more than a woman, drag queen, *meisie* (girl), feminine, “mable”, bottom	Around friends, in social gatherings (clubs & shebeens), in sexual interactions and relationships	With family members
Stacey Benjamin Martins	*meisie* (girl/woman), *moffie*, feminine, femgay, gay, female, woman, bottom	Around friends, social gatherings at clubs and shebeens, in sexual interactions and relationships	In new relationships having to negotiate her biological anatomy, with extended family members and community members
Conry Curella Jenkings	Gay, *meisie* (girl), femgay, queen (as part of the pagent/modelling shows), drag queen, *vroumens* (woman), male, “mable”, feminine, bottom	Cross-dressing and feminine identity in public with peers and friends, cross-dressing at home, in sexual interactions and relationships	On formal documentation and accessing services: *“we don’t ever say we’re males (laughs) unless you want to really know or you have my ID in hand”*, at school, initial and potential partners (having to explain assigned sex at birth)
Girlie Gregory Jones	*meisie* (girl), man, woman, gay	In sexual interactions and relationships (with her partner)	In romantic or sexual relationships – sometimes needing to be tough to counteract violence, on formal documentation, with her family
Georgina George Samuels	Woman, *moffie*, feminine pronouns, “vrou”/woman, man	Dresses and express feminine with family, family who acknowledges she has feminine hormones, in sexual interactions and relationships	Outside of regular social circles: clinics, work, shops, community; family also uses masculine name and pronouns to refer to her; with masculine/male friends
Sizwe Thafeni	Gay, *moffie*, MSM, lady/woman, male (but does not identify with the term), bottom	In sexual interactions and relationships; at work	Formal documentation (showing her ID document), with family members (like her mother)
Patricia Arends	Guy/man, woman, gay, *moffie, meisie* (girl)	In sexual interactions and relationships	Formal documentation (on ID document), community members, friends and household members

## Data Availability

The data used for this manuscript form part of a databank for the HPTN071 (PopART) study, which can be made available by application and request to the trial working group. Persons to contact regarding this are co-authors PB (in-country co-PI) and GH (socio-behavioural science lead and qualitive cohort design).

## Abbreviations

HIV: Human Immunodeficiency Virus
MSM: men who sex with men
PopART: Population Effects of Antiretroviral Therapy to Reduce HIV Transmission (PopART): A cluster-randomized trial of the impact of a combination prevention package on population-level HIV incidence in Zambia and South Africa
LGBTQI: Lesbian, Gay, Bisexual, Transgender, Queer and Intersex
